# Smart Flow Electrosynthesis and Application of Organodisulfides in Redox Flow Batteries

**DOI:** 10.1002/advs.202104036

**Published:** 2021-11-10

**Authors:** Qiliang Chen, Wei Guo, Yongzhu Fu

**Affiliations:** ^1^ College of Chemistry Zhengzhou University Zhengzhou 450001 P. R. China

**Keywords:** energy storage, organodisulfides, redox flow batteries, smart electrosynthesis

## Abstract

Electrochemical techniques have been recognized as an environmentally friendly and sustainable synthetic way to form organodisulfides. However, searching for optimum conditions which suffers from time/material‐consuming caused by the uncertainty of reactant consumption has hindered its rapid and large‐scale development. Inspired by advanced nonaqueous redox flow batteries (NARFBs) technology, it is proposed a smart flow electrosynthesis (SFE) method of organodisulfides that the voltage curve of NARFBs can be utilized as a precise indicator to reflect the desired information about reactants and distinguish the end point of reaction automatically. This electrochemical method also exhibits certain universality and scalability. Additionally, organodisulfides generated in electrolytes can be used as active species for NARFBs without further purification, and their electrochemical properties are easily adjusted by changing raw materials, which effectively alleviate the waste in complex synthesis steps for optimizing and designing active materials separately. An organodisulfide dervied from isopropyl alcohol and carbon disulfide shows excellent cycling life (1000 cycles) with low capacity fade rate (0.024% per cycle). Taking advantages of the inherent NARFBs, this work not only proves a SFE strategy, but also supplies a green and low‐cost molecular engineering scheme for designing electroactive materials for energy storage.

## Graphical abstract

Electrosynthesis in redox flow batteries is developed as a promising strategy for the synthesis of organodisulfides. It can also serve as a low‐cost molecular engineering scheme for designing organic electroactive molecules, because it can effectively alleviate the waste in complex synthesis steps for the separate optimization of active materials.

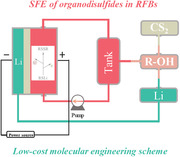

## 1 Introduction

**Figure 1:**
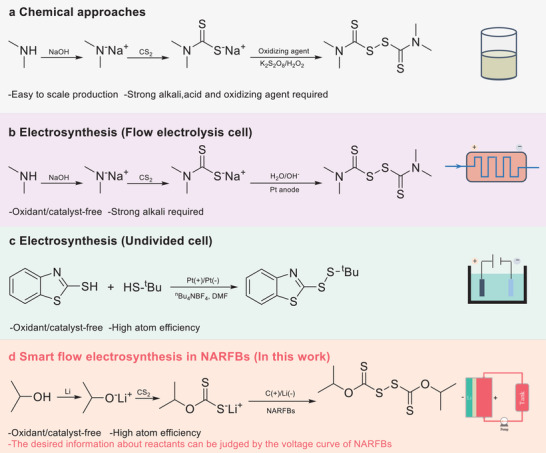
Strategies for synthesis of organodisulfides. a) Chemical approaches for synthesizing tetramethylthiuram disulfide. b) Electrosynthesis of tetramethylthiuram disulfide in flow electrolysis cell. c) Electrosynthesis of unsymmetrical disulfides in undivided cell. d) Smart flow electrosynthesis (SFE) of organodisulfides in NARFBs (Lithium:Li, Carbon:C).

Organodisulfides, containing disulfide (S—S) bonds, are important molecular motifs in pharmaceutical industry,^[^ ^1^ ^]^ life science,^[^ ^2^ ^]^ and energy storage^[^ ^3^ ^]^ owing to their unique pharmacological and physiochemical properties. Given their importance, various strategies have been developed for the synthesis of organodisulfides. Among them, chemical approaches remain the most oft‐applied methods. The use of unfriendly reagents (e.g., strong oxidant and alkali, **Figure** **1**a) has hindered its development in the modern era. Electrochemical strategies have emerged as a promising and environmentally friendly tool in organic synthesis, which possess many benefits over reagent‐based conversions, e.g., mild conditions and sustainability.^[^ ^4^ ^]^ Indeed, some electrochemical methodologies have been developed for green synthesis of organodisulfides (e.g., Figure 1b,c).^[^ ^5^ ^]^ Catalyst/oxidant‐free and less toxic‐water generated make these methods more advantageous in terms of synthetic sustainability and industrial safety. However, uncertainty of reactant consumption in the electrosynthesis processes results in the time/material‐consuming in search of optimum conditions, which has hindered its rapid and large‐scale development.^[^ ^6^ ^]^ Hence, development of a multifunction electrochemical tool that can not only enable electrosynthesis, but also monitor reactants and distinguish the end point of reactions automatically is highly desired.

As a promising energy storage technology for medium/large‐scales, nonaqueous redox flow batteries (NARFBs) integrate the merits of energy conversion and storage systems with high scalability.^[^ ^7^ ^]^ Electroactive materials dissolved in electrolytes are stored in outer tanks, which can flow through a connected electrochemical cell for chemical reactions.^[^ ^8^ ^]^ With the help of voltage changes in NARFBs, the chemical reactions could be monitored and stopped automatically. Perhaps we can take advantage of the unique properties of NARFBs to use in the electrosynthesis of organodisulfides. More importantly, if the organic electroactive molecules generated in the NARFBs can be directly used as active materials in the supporting electrolyte, it is beneficial for the development of low‐cost NARFBs.

Motivated from the above discussion, for the first time, we propose a smart flow electrosynthesis (SFE) method of organodisulfides based on the advanced NARFB technology (Figure 1d). Two types of benefits need to be noted. One is the NARFB as a smart strategy for flow electrosynthesis of organodisulfides. With the help of the voltage of NARFBs, the reaction status can be monitored, and automatically terminated in the absence of reactants, which avoid the time/material‐consuming in search of optimum conditions caused by the uncertainty of reactant consumption. Another is as green and low‐cost molecular engineering scheme for designing electroactive materials. Organodisulfides, generated in electrolytes, without any treatment can be used as electroactive species for NARFBs, and their electrochemical properties are easily adjusted by types of raw materials, which avoid unnecessary consumption caused by the preparation of organodisulfides alone. So, this strategy is very beneficial for the design and optimization of electroactive materials for energy storage.

## 2 Results and Discussion

### 2.1 SFE of Organodisulfides

**Figure 2:**
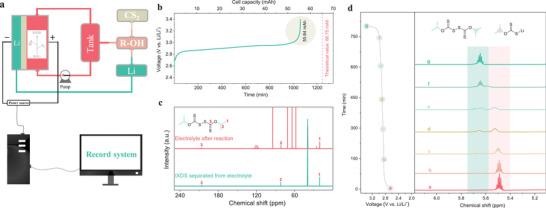
a) Schematic illustration of a SFE system. b) Voltage changes recorded in the electrosynthesis of IXDS in NARFBs at a current density of 1 mA cm^−1^ (electrolyte of 5 mL, flow rate of 12 mL min^−1^). c) ^13^ C NMR spectra of IXDS before and after separated from the electrolyte. d) Voltage profiles (left) and its corresponding a characteristic ^1^H NMR spectra (right) of IXDX/Li‐IX at different reaction stages (current density of 1 mA cm^−1^, electrolyte of 4 mL, flow rate of 12 mL min^−1^). All the reaction was performed with 0.5 m substrate dissolved in the supporting electrolyte.

The voltage curve of NARFBs reflects the mutual conversion between electric energy and chemical energy. At the end of the voltage curve, the large voltage difference caused by the concentration polarization between electrode surface and electrolyte indicates the completion of the conversion between electric energy and chemical energy. Besides this, organic electrolytes containing electroactive materials are stored in outer tanks (**Figure** **2**a), which can flow through a connected electrochemical cell for chemical reactions, and they are also a distinct advantage for large‐scale energy storage over than other types of batteries.^[^7f, 9^]^ Inspired by these characteristics of NARFBs, we sought to use NARFBs as a flow electrochemical tool to synthesize organodisulfides (Figure 2a). Besides some advantages of other types flow electrolysis cells with high conversions and selectivity,^[^ ^10^ ^]^ the voltage curve in NARFBs can be as a precise indicator to assess the formation of organodisulfides. Digital images of a complete SFE system and the flow cell components based on schematic diagram (Figure 2a) are provided in Figures S1 and S2 (Supporting Information), which consist of a NARFB (tank, pump, and electrochemical cell), battery testing system (power supply) and a computer with NEWARE BTS7.6.0 software (record system). The main division of labor in the electrosynthesis is as follows, raw molecules or target molecules are stored in outer tanks, which flow into or out of the electrochemical cell by a pump. Target molecules are generated in the electrochemical cell by electric energy supplied from battery testing system. The software is used to record the voltage curve and control the output and stop of power supply.

Isopropylxanthic disulfide (IXDS), an important molecule in vulcanization accelerators, flotation agent, and energy materials,^[^ ^11^ ^]^ was chosen to explain the SFE process. Typical synthesis process for IXDS involves two steps (Figure S3, Supporting Information): 1) a condensation reaction of isopropanol with alkali‐metal hydroxide and carbon disulfide (CS_2_) to form sodium isobutyl xanthate 2) which was oxidized by oxidizing agents (e.g., H_2_O_2_, K_2_S_2_O_8_, and I_2_).^[^ ^12^ ^]^ Inevitable side effects and strong oxidants are required leading to its less environmental friendliness.^[^ ^13^ ^]^ Based on the consideration of the formation process of IXDS and the characteristics of NARFBs, we modified the typical synthesis process and introduced it into NARFBs (Figure 1d). Isopropanol was first dehydrogenated by lithium (Li) metal and reacted with CS_2_ in the tank of NARFBs to produce lithium isobutyl xanthate (Li‐IX), and then it was converted to IXDS in the electrochemical cell of NARFBs. Besides the advantages of other electrosynthesis, some new desirable advantages originated from NARFBs need to be mentioned. As shown in Figure 2b, the voltage increases slowly in the initial reaction indicating that the supporting electrolyte contains many reactants, and the rise trend increases in the later stage prefiguring the reaction is coming to an end caused by the concentration polarization between electrode surface and electrolyte (Figure 2b, dark khaki area). A cutoff voltage can be set in the record system (Figure 2a; and Figure S1, Supporting Information) according to the character of IXDS to stop the electrosynthesis automatically. Furthermore, the efficiency of conversion from Li‐IX to IXDS can be acquired by the value of cell capacity, e.g., the theoretical value of a cell capacity is 66.75 mAh and the achieved value is 55.64 mAh (Figure 2b), so the efficiency of conversion for Li‐IX is 83.4% like using it to evaluate material utilization in NARFBs.^[^7d, 14^]^ The ^13^C nuclear magnetic resonance (NMR) spectra (Figure 2c) confirm the structure of IXDS before and after separation in the supporting electrolyte, and the other peaks from the supporting electrolyte and dimethyl sulfoxide (DMSO) (Figure S4, Supporting Information). To further comprehend the SFE, ^1^H NMR spectra were selected to illustrate the relationship between voltage variation and Li‐IX/IXDS concertation at different reaction stages. The full and a characteristic ^1^H NMR spectrum of Li‐IX/IXDS are presented in Figure 2d; and Figure S5 (Supporting Information). With the increase of voltage (Figure 2d, left), the peak value of Li‐IX disappears gradually, and the peak value of IXDS appears gradually. After the reaction stops, only the peak value of IXDS can be seen without the peak value of Li‐IX (Figure 2d, right, line e), suggesting that the end point of electrosynthesis process was accurately judged by the voltage curve of NARFBs.

### 2.2 Universality and Scalability for the SFE

**Figure 3:**
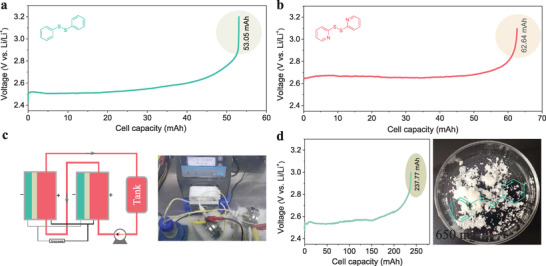
a) SFE of PhSSPh (current density of 1 mA cm^−1^, electrolyte of 5 mL, flow rate of 12 mL min^−1^). b) SFE of PySSPy (current density of 1 mA cm^−1^, electrolyte of 5 mL, flow rate of 12 mL min^−1^). c) Schematic illustration (left) and photograph (right) of a parallel SFE. d) Gram‐scale of SFE of PhSSPh (current density of 1 mA cm^−1^, electrolyte of 20 mL, flow rate of 12 mL min^−1^). All the reaction was performed with 0.5 m substrate dissolved in the supporting electrolyte.

To prove the SFE with certain universality and scalability, phenyl/pyridine disulfides (PhSSPh/PySSPy) derived from thiols (thiophenol/2‐pyridinethiol) were synthesized in NARFBs. The phenomenon of the voltage changes recorded in the electrosynthesis of PhSSPh/PySSPy (**Figure** **3**a,b) is similar to the above. The ^13^C and ^1^H NMR spectra confirm the formation of disulfides, the characteristic peaks of PhSSPh/PySSPy are observed in Figure S6 (Supporting Information), and there are basically no peaks of lithium benzenethiolate/lithium pyridine‐2‐thiolate in the ^13^C/^1^H NMR spectra of PhSSPh/PySSPy. As in the design of NARFBs, the production rate of organodisulfides is affected by the reaction surface area, and the production quantity of organodisulfides is limited by the size of tank. So, paralleling multiple NARFBs or directly increasing the electrode size of a single NARFB could improve the production rate of organodisulfides. Based on this, we put two flow cells together in parallel to obtain a SFE system with double reaction area (Figure 3c). Gram scale of SFE of PhSSPh was achieved (Figure 3c, right), and the voltage changes are shown in Figure 3d with a high conversion efficiency of 88.8% based on the ratio of the obtained cell capacity/theoretical capacity of PhSLi in the cell. The as‐synthesized PhSSPh can be easily separated from the electrolyte (Figure 3d, right; and Figure S7, Supporting Information with a yield of 67.1% due to its hydrophobicity. ^13^C and ^1^H NMR spectra confirm the structure of PhSSPh (Figure S8, Supporting Information), excluding the peaks from DMSO, the other peaks observed are completely consistent with those of PhSSPh. the advanced NARFB technology as a novel strategy to electrochemically synthesize organodisulfides has certain universality and scalability, which effectively avoid the potential waste of time and material caused by searching the optimum conditions, especially in large‐scale production.

### 2.3 Application of Organodisulfides in NARFBs

For NARFBs, electroactive materials play a critical role in the electrochemical performance of NARFBs, including energy density, power density, cycle life, efficiency, capital cost, and ecological footprint.^[^ ^15^ ^]^ The well‐developed organic synthesis techniques have become a mainstream path to design and modify electroactive materials to optimize their redox potential, solubility, and molecular size.^[^ ^16^ ^]^ Indeed, some molecules with ideal electrochemical behavior in NARFBs can be synthesized by these strategies. Despite many progresses, the prices of electroactive materials not only reply on the precursors, but also are greatly affected by the complex synthesis steps and separation processes, leading to high cost of NARFBs. Therefore, organic electroactive molecules, generated in the NARFBs directly, could effectively alleviate the waste in complex synthesis steps and may lead to low cost of the devices.

**Figure 4:**
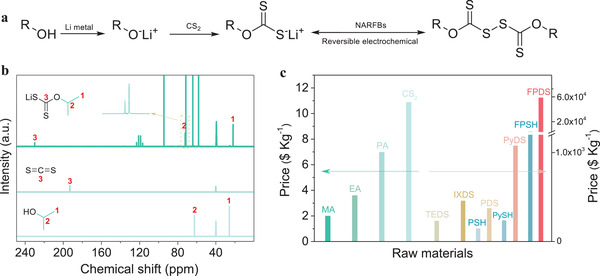
a) SFE and application of xanthogen disulfides in NARFBs. b) ^13^C NMR spectra of Li‐IX, carbon disulfide and isopropanol. c) Comparison the prices of alcohols/carbon disulfide and some representative organodisulfides. (Methanol: MA, Ethanol: EA, Isopropanol: PA, Tetraethylthiuram disulfide: TEDS, Benzenethiol: PSH, Phenyl disulfide: PDS, 2‐Pyridinethiol: PySH, 2,2′‐dipyridyl disulfide: PyDS, 3‐Fluorothiophenol: FPSH, and Bis(3‐fluorophenyl) disulfide: FPDS)

Based on the above considerations, three xanthate salts (lithium methyl xanthate: Li‐MX, lithium ethyl xanthate: Li‐EX, and lithium isopropyl xanthate: Li‐IX) derived from alcohols (methanol, ethanol, and isopropanol) were synthesized in the supporting electrolyte (**Figure** **4**a). ^13^C and ^1^H NMR spectra confirm the structure of xanthate salts (see Figure 4b; and Figures S9 and S10, Supporting Information), excluding the peaks from the supporting electrolyteor DMSO, the other peaks observed are completely consistent with the result of xanthate salts. Apart from that, there are basically no peaks of alcohols and CS_2_ in the ^13^C/^1^H NMR spectra of xanthate salts. And strong peaks at about 1650 cm^−1^ are observed in the infrared spectra of xanthate salts (see Figure S11, Supporting Information), which are assigned to the C═S groups. The above results indicate that xanthate salts can be well prepared in the supporting electrolyte. More noteworthy, the outstanding price advantages of alcohols, CS_2_, and thiols compared with commercial IXDS and other representative organodisulfides are shown in Figure 4c; and Table S1 (Supporting Information), which greatly reduce the specific cost proportions of electroactive materials in NARFBs (Figure S12, Supporting Information).

**Figure 5:**
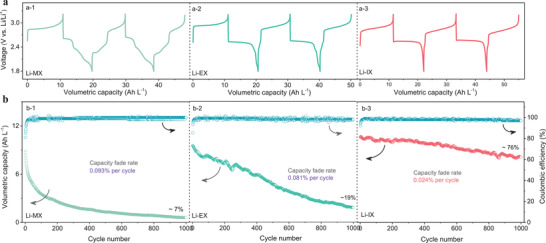
a) Representative charge and discharge profiles of xanthate salts at a current density of 0.5 mA cm^−2^. b) Cycling performance of xanthate salts at 0.5 mA cm^−2^.

The galvanostatic charge and discharge were performed to evaluate the electrochemical performance of 0.5 m xanthate salts in NARFBs. During the first cycle of charge, similar voltage plateaus are observed in three xanthate salts (**Figure** **5**a), meaning that the formation of disulfide compounds. Long‐term cycling of 0.5 m Li‐IX with a high capacity retention rate of 76% is achieved for 1000 cycles at 0.5 mA cm^−2^, which is higher than those of Li‐MX and Li‐EX, i.e., 7% and 19%, respectively. The reason for this result may be due to the instability of xanthogen disulfides (MXDS and EXDS) in the supporting electrolyte. To prove this, xanthogen disulfides (EXDS and IXDS) derived from ethanol and isopropanol were synthesized in NARFBs, and the electrolytes stood for 1 week. The electrolyte containing EXDS shows a lot of precipitation (Figure S13a, Supporting Information) and lower discharge capacity (3.1 Ah L^−1^) than IXDS (9.7 Ah L^−1^) (Figure S13b, Supporting Information), indicating that the EXDS is unstable in the supporting electrolyte. Furthermore, achieving a long cycling life at high concentrations of electroactive materials is meaningful for practical applications in NARFBs.^[7d]^ Posolyte with 1.0/2.0 m Li‐IX were successfully obtained (Figure S14, Supporting Information), which deliver first discharge reversible capacities of 17.4 and 32.5 Ah L^−1^ with 80% (400 cycles for 1.0 m) and 74% (200 cycles for 2.0 m) capacity retentions, respectively. This synthesis strategy has been proved to be a sustainable and low‐cost molecular engineering method for the development of advanced energy storage molecules.

## 3 Conclusion

In summary, we propose a SFE method of organodisulfides inspired from the advanced NARFB technology. The voltage curve of NARFBs serves as a precise indicator to reflect the desired information about reactants and distinguish the end point of reaction automatically, which has certain universality and scalability and greatly avoids the time/material‐consuming for condition optimization. More notably, the electrolyte containing the as‐synthesized electroactive materials with different functional groups, without any purification, can be directly used for NARFBs. An organodisulfide dervied from isopropyl alcohol and carbon disulfide shows excellent cycling stability (1000 cycles with low capacity fade rate, 0.024% per cycle) and high Coulombic efficiency (97%). So, this strategy also provides us a sustainable and low‐cost molecular engineering scheme for designing organic electroactive molecules. We believe that inspiration from advanced RFB technologies will have an important impact in SFE of various organic molecules, especially some potential energy storage molecules.

## 4 Experimental Section

### Materials

Methyl alcohol (CH_3_OH, MA, 99.9%), ethanol (CH_3_CH_2_OH, EA, 99.5%), 2‐propanol (C_3_H_7_OH, PA, 99.5%), carbon disulfide (CS_2_, 99.9%), and 2‐pyridinethiol (C_5_H_4_NSH, 99%) were received from Adamas. Benzenethiol (C_6_H_5_SH) was received from Kina chemical. Dimethyl ether (DME, 99.95%), 1,3‐dioxolane (DOL, 99.95%), and Lithium bis(trifluoromethanesulfonyl)imide (LiTFSI, 99.8%) were received from DoDoChem. Commercial electrolytes were received from Canrd which consist of 1 m LiTFSI and 0.2 m lithium nitrate (LiNO_3_) in DME/DOL. Lithium metal was received from China Energy Lithium Co., LTD. Carbon paper was received from NanoTechLabs Composites, Inc. Graphite felt was received from Dalian Longtian Tech Co., Ltd with a thickness of about 3 mm.

### SFE Cell Assembly and Electrosynthesis of Organodisulfides

The assembled process of a SFE cell in the Ar‐filled glove box (H_2_O < 0.01 ppm, O_2_ < 0.01 ppm) as follows. First, a piece of lithium metal (Ø20 mm) was added into the anode side followed by adding 100 µL blank electrolyte, and then a Celgard separator (Ø25 mm) was placed on the surface of the lithium metal. For the cathode side, one piece of carbon paper (Ø20 mm) and one piece of graphite felt (Ø20 mm) were added into a polytetrafluoroethylene (PTFE) flow channel (Ø25*20*3.0 mm), and the effective reacting area of a single cell is 3.14 cm^2^.

For the electrosynthesis of isopropylxanthic disulfide, isopropanol (150.1 mg) was first lithated dehydrogenation by lithium metal (17.4 mg) in 5.0 mL DOL/DME (v:v = 1:1), and then react with CS_2_ (190.4 mg) to form lithium isobutyl xanthate, the last is to add lithium salts (717.6 mg LiTFSI/ 51.7 mg LiNO_3_) and flow into the electrochemical cell for chemical reaction. For the electrosynthesis of phenyl disulfide and pyridine disulfide, the molar ratio of thiols and lithium metal is 1:1, benzenethiol (275.4 mg for 5.0 mL electrolyte, 1101.7 mg for 20.0 mL electrolyte) and 2‐pyridinethiol (277.9 mg) are lithated dehydrogenation by lithium metal (17.4 or 69.4 mg) directly in commercial electrolytes, respectively, then flow into the electrochemical cell for chemical reaction. All the electrolyte (5.0 or 20 mL) was circulated between the tank and the electrochemical cell at 12.0 mL min^−1^ by a peristaltic pump (Lead Fluid).

### Purification Process

For PhSSPh, the reaction solution containing PhSSPh was added into water, and after standing for 24 h, PhSSPh precipitated in the solution. Finally, PhSSPh was obtained after filtration and multiple washing by water. For IXDS, its purification process is similar to that of PhSSPh due to its hydrophobicity. After the separation, the purity of IXDS and PhSSPh is ≈94% and ≈98%, respectively.

### Assembly of Static Battery

The assembled process of a static battery in the Ar‐filled glove box (H_2_O < 0.01 ppm, O_2_ < 0.01 ppm) as follows. Briefly, 15 *μ*L catholyte was dropped onto a carbon paper (Φ = 12 mm), and then a piece of Celgard 2400 (Φ = 19 mm) was pressed onto the carbon paper. Next, 20 *μ*L commercial electrolyte was added to the anode part, and a piece of lithium (Φ = 16 mm) was placed on the electrolyte.

### Electrochemical Measurements

A battery testing system (Neware, BTS‐5V) was used to supply electricity. The cut‐off voltage for smart electrosynthesis of organodisulfides was set to 3.0 V or greater at the current density of 1.0 mA cm^−2^. For the static battery, the cut‐off voltage was set to 1.8–3.2 V at 0.5/1.0 mA cm^−2^.

### Characterization

The ^1^H and ^13^C NMR spectrum were recorded on a Bruker Ascend TM 600 MHz , and all the samples were dissolved in DMSO. The Fourier transform infrared (FT‐IR) spectra were recorded in the range 400–4000 cm^−1^ on Bruker Alpha P spectrometer with a single reflection ATR.

## Conflict of Interest

The authors declare no conflict of interest.

## Supporting information

Supporting Information

## Data Availability

The data that support the findings of this study are available from the corresponding author upon reasonable request.
