# Supplementary material for: Smart Flow Electrosynthesis and Application of Organodisulfides in Redox Flow Batteries
Source: Adv Sci (Weinh). 2021 Nov 10;9(1):2104036. doi: 10.1002/advs.202104036 (PMC8728815; doi:10.1002/advs.202104036)
Supplement: Supplementary file 1 — Supporting Information [file ADVS-9-2104036-s001.pdf]

WILEY-VCH

© 2021 Wiley-VCH GmbH

ADVANCED SCIENCE
Open Access

# Supporting Information

for *Adv. Sci.*, DOI: 10.1002/advs.202104036

# Smart flow electrosynthesis and application of organodisulfides in redox flow batteries

*Qiliang Chen, Wei Guo, and Yongzhu Fu**

# Supporting Information

## Smart flow electrosynthesis and application of organodisulfides in redox flow batteries

*Qiliang Chen, Wei Guo,and Yongzhu Fu**

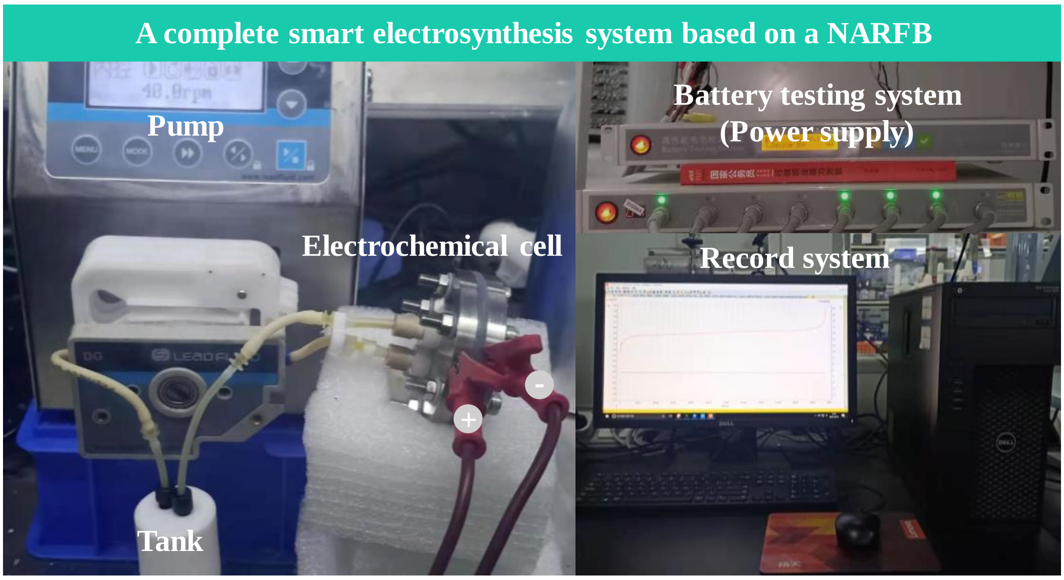


**Figure S1.** Digital images of a NARFB for SFE of organodisulfides.

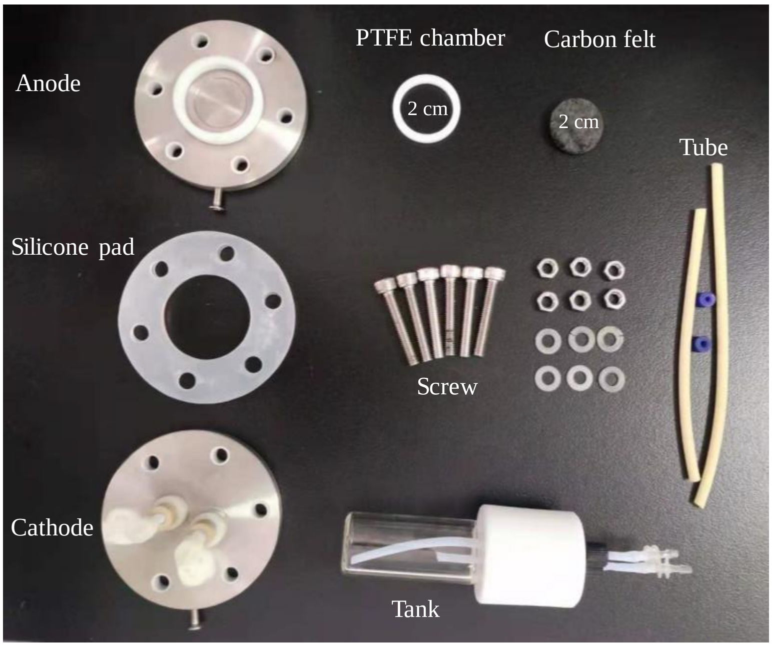


**Figure S2.** Digital images of a RFB components.

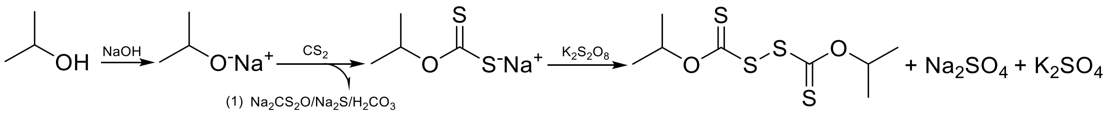


**Figure S3.** A typical chemical synthesis process of isopropylxanthic disulfide (IXDS).

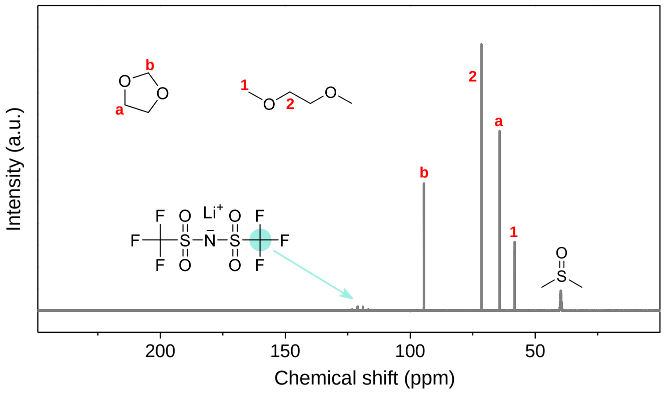


**Figure S4.** $^{13}$C NMR spectra of the blank supporting electrolyte, which contains of solvent (DME and DOL) and lithium salt (LiTFSI).

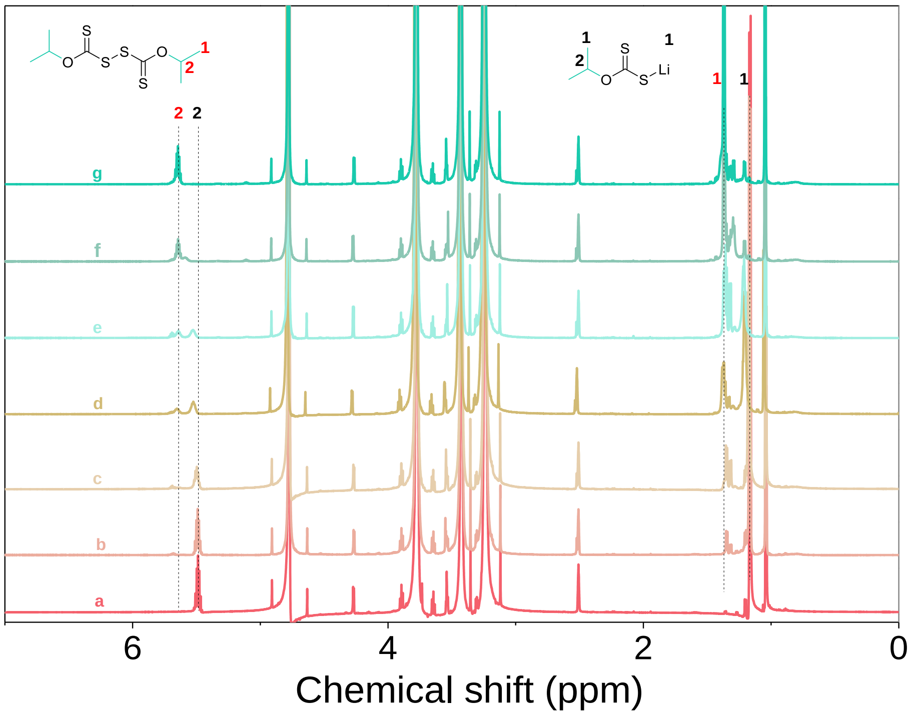


**Figure S5.** $^{1}$H NMR spectra of the reaction solution at different stages for the smart flow electrosynthesis of IXDS in NARFBs. (current density of 1 mA cm$^{-1}$, electrolyte of 4 mL, flow rate of 12 mL min$^{-1}$, the reaction was performed with 0.5 M Li-IX dissolved in the supporting electrolyte)

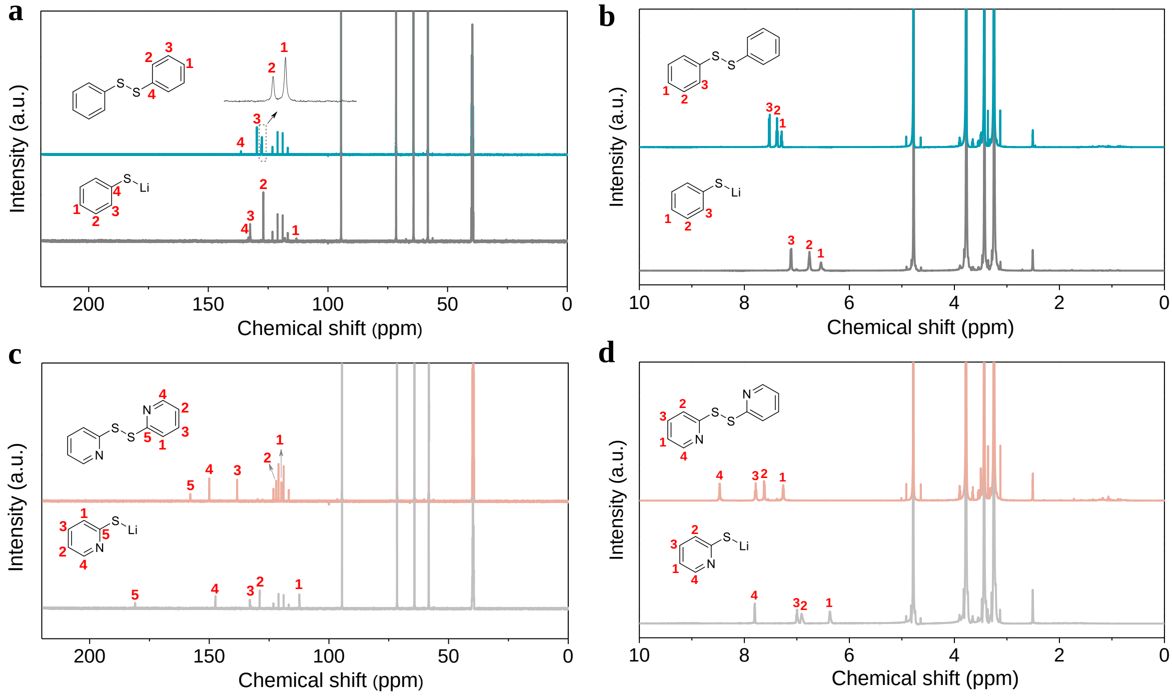


**Figure S6. (a)** $^{13}C$ NMR spectra of PhSSPh and lithium benzenethiolate. **(b)** $^{1}H$ NMR spectra of PhSSPh and lithium benzenethiolate. **(c)** $^{13}C$ NMR spectra of PySSPy and lithium pyridine-2-thiolate. **(d)** $^{1}H$ NMR spectra of PySSPy and lithium pyridine-2-thiolate.

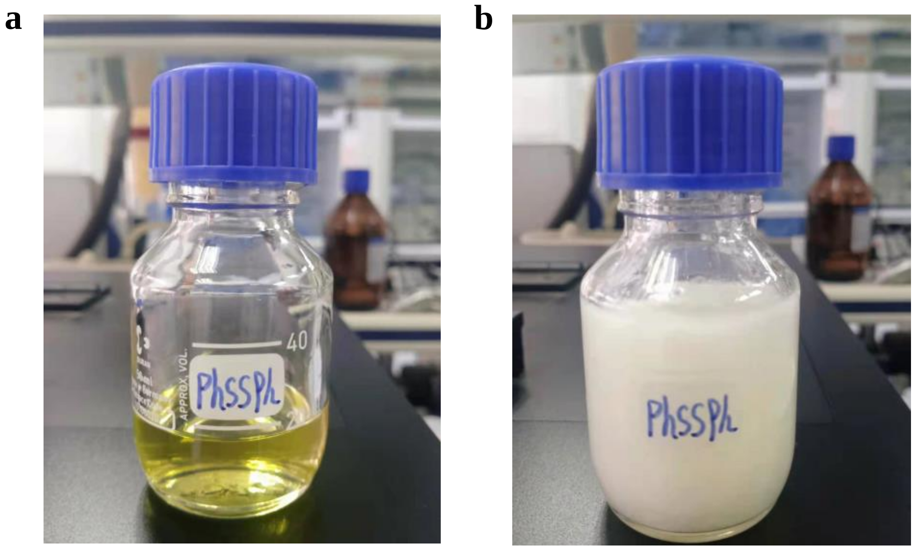


**Figure S7.** Digital images of the electrolyte for the gram-scale of smart flow electrosynthesis of PhSSPh before (**a**) and after (**b**) adding water, and its corresponding voltage profiles seen in Fig.3d. (current density of 1.0 mA $cm^{-1}$, electrolyte of 20 mL, flow rate of 12 mL $min^{-1}$).

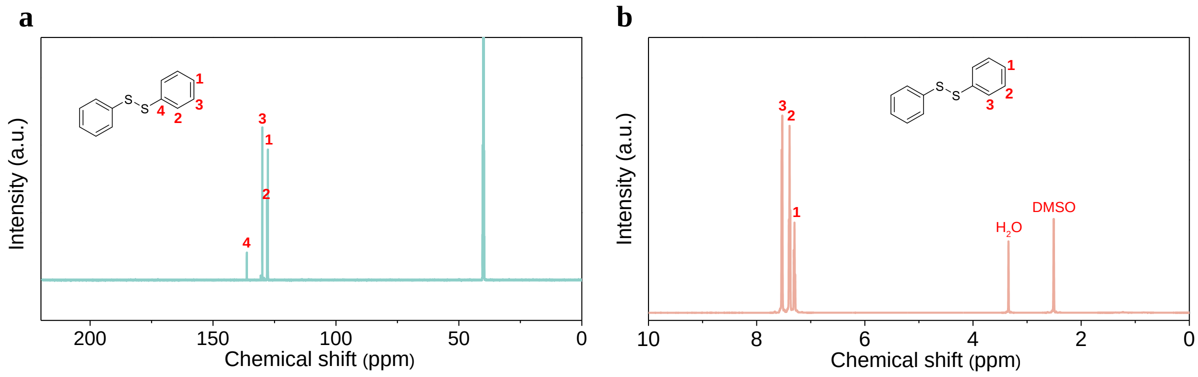


**Figure S8.** $^{13}C$ and $^{1}H$ NMR spectra of PhSSPh after separation from the supporting electrolyte, and its corresponding optical images for pure PhSSPh seen in Figure 3d (right).

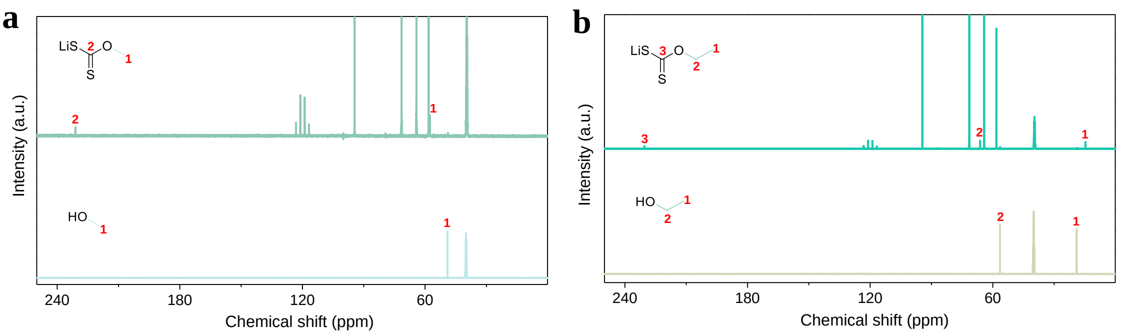


**Figure S9.** $^{13}C$ NMR spectra. (**a**) Li-MX and MA. (**b**) Li-EX and EA.

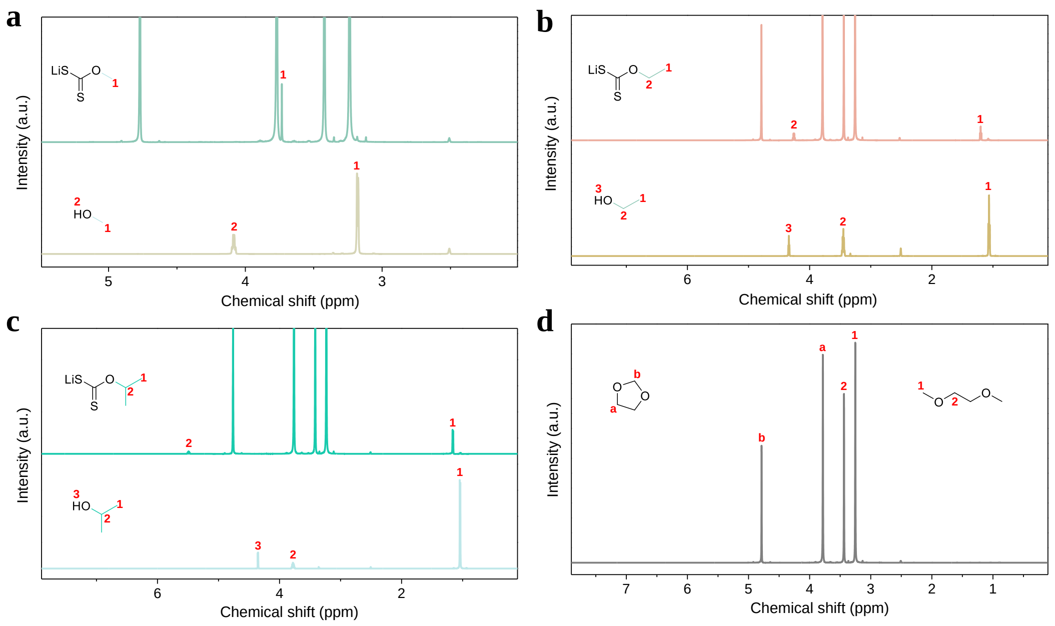


**Figure S10.** **$^{1}H$ NMR spectra**. **(a)** Li-MX and MA. **(b)** Li-EX and EA. **(c)** Li-IX and PA. **(d)** Blank supporting electrolyte.

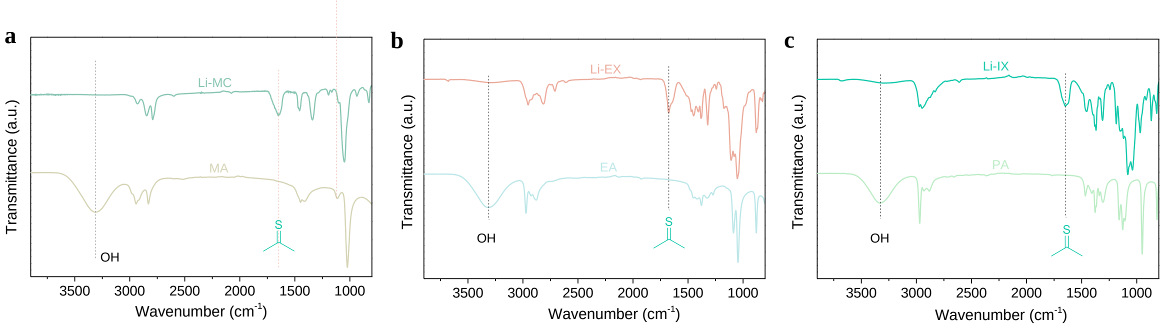


**Figure S11.** FT-IR spectrum. **(a)** Li-MX and MA. **(b)** Li-EX and EA. **(c)** Li-IX and PA.

**Table 1.** The prices of raw materials

| Raw materials | Price \$ kg$^{-1}$ |
|---|---|
| Methyl alcohol | 2.0 |
| Ethanol | 3.6 |
| 2-propanol | 7.0 |
| carbon disulfide | 10.9 |
| 2-pyridinethiol | 238.3 |
| Benzenethiol | 147.2 |
| 3-fluorothiophenol | 3945.0 |
| Isopropylxanthic disulfide | 461.0 |
| Tetraethylthiuram disulfide | 234.0 |
| Phenyl disulfide | 375.0 |
| 2,2'-dipyridyl disulfide | 1078.9 |
| Bis(3-fluorophenyl) disulfide | 58912.8 |

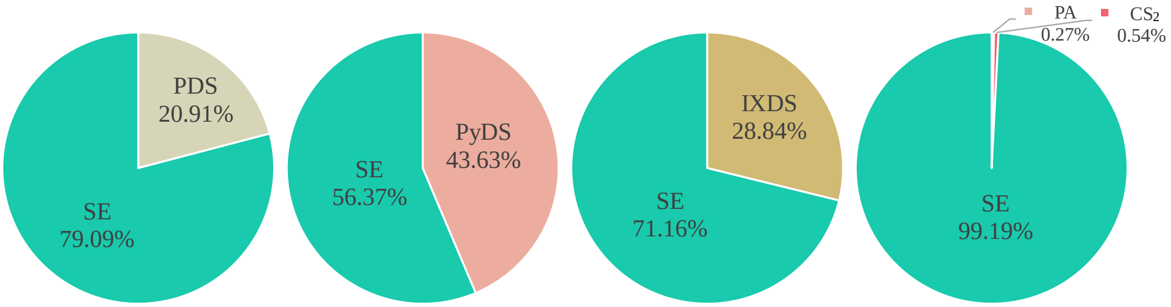


**Figure S12.** Comparison of the cost specific proportions of 1.0 M organodisulfides in catholyte for NARRBs. (SE: Supporting electrolyte, PDS: Phenyl disulfide, PyDS:2,2'-dipyridyl disulfide, IXDS: Isopropylxanthic disulfide, PA: Isopropanol). The price of SE is obtained from canrd. And The prices of organodisulfides and other materials are obtained from www.tansoole.com or www.shyuanye.com.

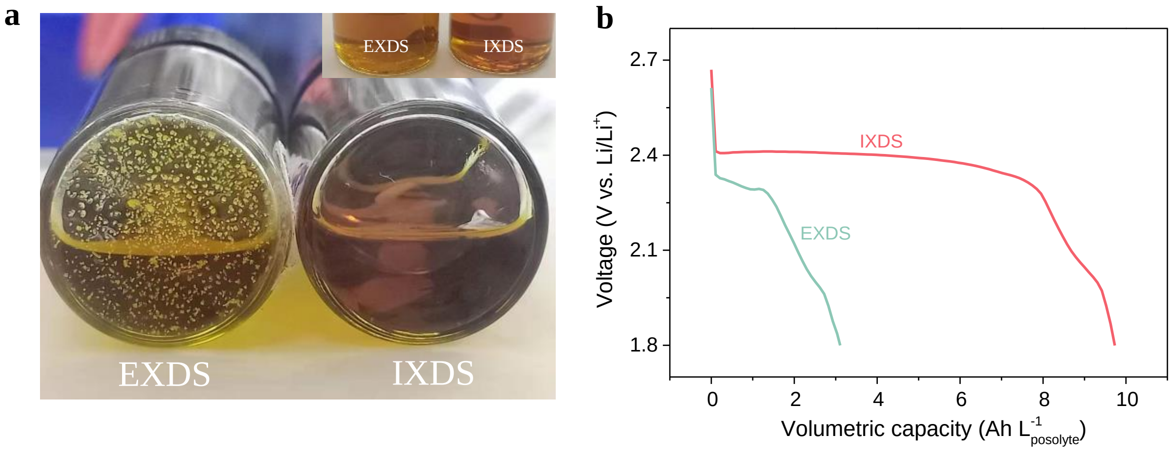


**Figure S13. (a)** Photographs of EXDS and IXDS in the electrolyte after being shelved for a week. **(b)** Corresponding to the discharge curve of the supernatant in **a**.

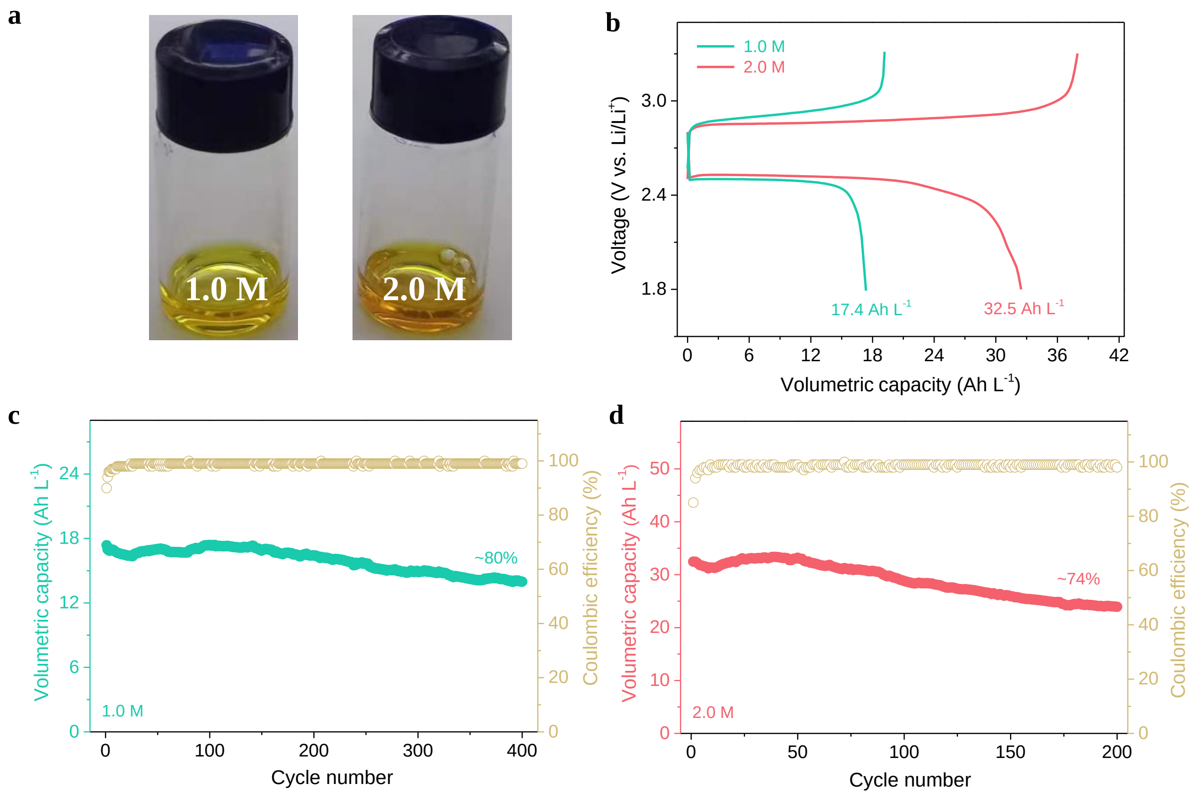


**Figure S14.** **(a)** Photographs of 1.0 and 2.0 M Li-IX in the supporting electrolyte. **(b)** The first charge/discharge curves of 1.0 and 2.0 M Li-IX. Cycling performance of 1.0 M (**c**) and 2.0 M (**d**) Li-IX at 1.0 mA $cm^{-2}$.
